# Predictors of physical activity promotion in clinical practice: a cross-sectional study among medical doctors

**DOI:** 10.1186/s12909-022-03686-z

**Published:** 2022-08-17

**Authors:** Catarina Santos Silva, Romeu Mendes, Cristina Godinho, Ana Monteiro-Pereira, Jaime Pimenta-Ribeiro, Helena Silva Martins, João Brito, José Luís Themudo-Barata, Carlos Fontes-Ribeiro, Pedro J. Teixeira, Graça Freitas, Marlene Nunes Silva

**Affiliations:** 1 CIPER, Faculdade de Motricidade Humana, Universidade de Lisboa, Lisbon, Portugal; 2 Programa Nacional para a Promoção da Atividade Física, Direção-Geral da Saúde, Lisbon, Portugal; 3 EPIUnit – Instituto de Saúde Pública, ITR, Universidade do Porto, Porto, Portugal; 4 Portugal Football School, Portuguese Football Federation, Cruz Quebrada, Portugal; 5 ACES Douro I - Marão e Douro Norte, Administração Regional de Saúde do Norte, Vila Real, Portugal; 6 Católica Research Centre for Psychological, Family and Social Wellbeing, Universidade Católica Portuguesa, Lisbon, Portugal; 7 CIDESD, University Institute of Maia, Maia, Portugal; 8 ACES Loures-Odivelas, Administração Regional de Saúde de Lisboa e Vale do Tejo, Lisbon, Portugal; 9 ACES Baixo Vouga, Administração Regional de Saúde do Centro, Aveiro, Portugal; 10 Serviço de Nutrição e Atividade Física do Centro Hospitalar Universitário da Cova da Beira, Covilhã, Portugal; 11 Faculdade de Ciências da Saúde da Universidade da Beira Interior, Covilhã, Portugal; 12 Instituto de Farmacologia e Terapêutica Experimental, Subunidade 1, Faculdade de Medicina, Universidade de Coimbra, Coimbra, Portugal; 13 Direção-Geral da Saúde, Lisbon, Portugal; 14 CIDEFES, Faculdade de Educação Física e Desporto, Universidade Lusófona, Lisbon, Portugal

**Keywords:** Medical doctors, Physical activity promotion, Predictors, Physical activity behaviours, Attitudes, Knowledge, Barriers, Confidence, Training, Opportunities

## Abstract

**Background:**

Physical activity is a major determinant of physical and mental health. International recommendations identify health professionals as pivotal agents to tackle physical inactivity. This study sought to characterize medical doctors’ clinical practices concerning the promotion of patients’ physical activity, while also exploring potential predictors of the frequency and content of these practices, including doctors’ physical activity level and sedentary behaviours.

**Methods:**

A cross-sectional study assessed physical activity promotion in clinical practice with a self-report questionnaire delivered through the national medical prescription software (naturalistic survey). Physical activity and sedentary behaviours were estimated using the International Physical Activity Questionnaire (short form). Indicators of medical doctors’ attitudes, knowledge, confidence, barriers, and previous training concerning physical activity promotion targeting their patients were also assessed. Multiple regression analysis was performed to identify predictors of physical activity promotion frequency by medical doctors, including sociodemographic, attitudes and knowledge-related variables, and physical activity behaviours as independent variables.

**Results:**

A total of 961 medical doctors working in the Portuguese National Health System participated (59% women, mean age 44 ± 13 years) in the study. The majority of the participants (84.6%) reported to frequently promote patients’ physical activity. Five predictors of physical activity promotion frequency emerged from the multiple regression analysis, explaining 17.4% of the dependent variable (*p* < 0.001): working in primary healthcare settings (*p* = 0.037), having a medical specialty (*p* = 0.030), attributing a high degree of relevance to patients’ physical activity promotion in healthcare settings (*p* < 0.001), being approached by patients to address physical activity (*p* < 0.001), and having higher levels of physical activity (*p* = 0.001).

**Conclusions:**

The sample of medical doctors approached reported a high level of engagement with physical activity promotion. Physical activity promotion frequency seems to be influenced by the clinical practice setting, medical career position and specialty, attitudes towards physical activity, and perception of patients´ interest on the topic, as well as medical doctors’ own physical activity levels.

## Introduction

Despite the growing evidence placing physical activity as a major determinant of population’s physical and mental health [1, 2], countries around the world face serious challenges concerning its promotion [3, 4]. According to the Global Action Plan for Physical Activity 2018–2030 [5] by the World Health Organization (WHO), a joint and intersectoral approach is needed to tackle physical inactivity. Within the healthcare sector, the WHO recommends the establishment of systems for patient assessment and counselling on increasing physical activity and reducing sedentary behaviour, implemented by appropriately trained health professionals, as a priority action in this regard [5, 6].

Healthcare sector offers unique conditions to promote population’s physical activity. First, it provides a direct contact with larger segments of population throughout the life course [7], as most people visit their medical doctor at least once annually [8]. Hence, individual-centred care, including opportunistic risk stratification, tailored advice, and behaviour change support can be ensured. Second, public trust in the healthcare sector increased during the last half decade [9]. More physically active healthcare providers seem to be in a particularly good position, as they tend to counsel physical activity more frequently to their patients than their less active counterparts, and can possibly act as role models [10–12]. Studies on physical activity promotion interventions implemented in healthcare settings, namely in primary healthcare, have also gathered evidence for effectiveness and cost-effectiveness [13–17]. Given that small increases in physical activity can lead to significant health benefits [18, 19], even a low to moderate efficacy of these interventions at the individual level can have a large impact on public health [20].

Knowing that medical doctors are considered essential players in physical activity counselling and promotion [21], different governmental entities rely on them to improve physical activity in their communities, with several programs being launched at the national and international levels [3, 5, 19]. However, several barriers have been highlighted: limited consultation time, absence of unambiguous protocols [22], insufficient knowledge and confidence due to insufficient training during the pre-graduate medical curricula [23, 24], and the perception that patients are ambivalent or have little motivation to change their behaviour [3, 5, 8, 25, 26]. As active medical doctors also seem to promote patients’ physical activity more frequently [12], having a better understanding of their own movement behaviours can help understanding and foster clinical practice.

Available data on the prevalence of physical activity promotion in primary healthcare reveals a wide variation for both physical activity assessment and counselling [27]. Concerning physical activity assessment, data from medical chart audits shows that 2.4 to 60.1% of primary healthcare patients had their physical activity levels assessed, and 8 to 100% of medical doctors reported to assess physical activity, at least, in some of their patients. Regarding physical activity counselling, 0.6 to 100% of medical doctors report advising physical activity to their patients, although only 1.5 to 52.2% of patients were given physical activity counselling (data from medical chart audits) [26]. In Portugal, in a representative sample of adults from a national survey in 2017, from those who had a medical appointment in the previous 2 years, 41% reported being advised to increase physical activity by their medical doctor [28]. At that time, there was no formal health policy being implemented in the Portuguese National Health Service (NHS) regarding physical activity promotion.

Understanding the reality of each country concerning physical activity promotion in healthcare settings is crucial for enhancing health outcomes in the future. It is important to describe the current medical doctors’ clinical practices for the promotion of their patients’ physical activity and barriers faced, as well as the determinants of these practices (i.e., attitudes, knowledge, own physical activity levels). Integrated monitoring systems at the national level are needed to provide information on these indicators, to understand their trends and to contribute for the continuous adjustment and improvement of public health policies in this area.

This study aimed to (i) describe physical activity promotion practices by medical doctors working in the Portuguese healthcare system; (ii) explore predictors of the frequency and content of these practices, including (iii) assessing the level of physical activity and sedentary behaviours of medical doctors.

## Methods

### Study design and setting

This was a cross-sectional study, based on an online survey disseminated via the Electronic Medical Prescription software (PEM), available to medical doctors working in the Portuguese health system. Data collection was carried out between January 19 and February 3, 2018. The study was coordinated by the National Program for Physical Activity Promotion from the Portuguese Directorate-General of Health, in collaboration with the Shared Services of the Ministry of Health (SPMS).

The study protocol was submitted and approved by the Ethics Committee and by the Scientific Council of the Faculty of Medicine of the University of Coimbra (Code 117, November 27, 2017), and complied with the principles of the Declaration of Helsinki [29]. Before enrolment, participants were informed about the purposes and procedures of the study and gave their informed consent, via the online information sheet that accompanied the online survey. Participation did not involve financial incentives.

### Participants and recruitment

This study was based on the responses of a self-selected sample of medical doctors working in the Portuguese health system. A required sample size of 379 participants was previously calculated with the Epi Info® software, considering a 95% confidence level and expected response rate of 50%. According to SPMS, on December 31, 2017, there were 57,922 registered medical doctors in Portugal, and 29,954 were PEM software users. The questionnaire was promoted electronically via PEM software (a pop-up window was automatically distributed for users).

Individuals under the age of 23 (due to the minimum required period of academic training), and over 69 [due to age recommendations for the analysis of International Physical Activity Questionnaire (IPAQ)-related questions [30, 31]], were excluded from the analysis.

### Measures

#### Demographics

Participants reported their birth year, sex, medical career position, medical specialty, clinical practice setting and sector, and country’s health administrative region.

#### Clinical practice regarding physical activity promotion

Participants reported the frequency which they were approached by patients asking advice on physical activity – on a 5-point Likert-type scale ranging from “very high” to “very low”, and if they promote patients’ physical activity in their clinical practice (yes or no). Those who reported promoting patients’ physical activity were asked about the frequency of this practice on the same 5-point scale, the physical activity components usually covered (i.e., sedentary behaviour; active mobility; exercise and sports), the number of week days of physical activity usually recommended to patients, the usual recommended length and the type of physical activities most frequently recommended. Participants also reported if they usually ask for any diagnostic test, before advising physical activity and, if yes, which one(s) they usually asked for.

#### Attitudes toward physical activity promotion, provided in healthcare settings

On a 5-point Likert-type scale, ranging from *“very high”* to *“very low”*, participants indicated: (i) the relevance they consider physical activity promotion has in healthcare settings; (ii) the importance given to the presence of exercise professionals in healthcare settings.

#### Knowledge and confidence about physical activity promotion

Participants indicated the degree of certainty of knowing the current physical activity guidelines (namely, aerobic and resistance physical activity) for adults, older adults, and children and youth – again on a 5-point scale ranging from “very high” to “very low”. Using an 11-point scale, ranging from 0 to 10, participants indicated they self-perceived degree of confidence in promoting physical activity among different stages of life span and health conditions (e.g., healthy adults, children, elderly, cardiovascular disease, oncologic disease, etc.).

#### Barriers to physical activity promotion

Participants were asked to select the main perceived barriers in their clinical practice regarding physical activity promotion from a list. Options included lack of time, lack of technical knowledge, fear of risks, and lack of interest of the patients, or other barrier – to be described by the participant.

#### Physical activity promotion training

Participants reported if they have ever had any training in physical activity promotion (and of which kind) and how many hours of such training they had. They were also asked if they had interest in having training in physical activity promotion and, if yes, in which specific areas.

#### Physical activity levels and sedentary behaviours

Physical activity and sedentary behaviour (sitting time) were assessed using the short form of IPAQ [30–32]. Frequency and duration of total vigorous-intensity activities, moderate-intensity activities, and walking performed over the previous week in, at least, 10-min bouts were reported by the participants. Using the IPAQ scoring protocol [31], weekly physical activity volume and energy expenditure in metabolic equivalents (MET) were estimated and participants were categorized as having a “low”, “moderate”, or “active” [health-enhancing physical activity (HEPA)] level. Participants also reported time spent sitting on a typical day over the previous week and were classified according to three categories: ≤3 hours a day; > 3 to < 7 hours a day; ≥7 hours a day [33–35].

With the exception of the IPAQ-short form, the whole questionnaire was developed by a specialist panel of the National Physical Activity Promotion Program of the Directorate-General of Health, on behalf of the Portuguese Health Ministry. The questionnaire was pre-tested by 10 Family Medicine medical doctors, regarding comprehension of the questions and time of completion, which was, on average, 12 minutes.

### Statistical analyses

The statistical procedures were processed using SPSS® software (Version 27.0. Armonk, NY: IBM Corp). Variables were described using absolute (*n*) and relative (%) frequencies, median and percentile values. Birth year was used to calculate participants’ age (years) at questionnaire completion. Physical activity promotion frequency by medical doctors was categorized into four categories (never; very low or low; medium; and high or very high) converging the responses of two questions of the questionnaire: one asking if they promote patients’ physical activity in their clinical practice (yes or no) and, to those who reported “yes”, another one asking the frequency of this practice (very high; high; medium; low; or very low). To test whether there were significant differences in physical activity promotion frequency in relation to sociodemographic characteristics, tests of independence were performed using Chi-Square test. Age was categorized into three groups (23–39; 40–54; and 55–69 years) for descriptive and Chi-square analysis purposes, only. In the regression models, age was used as continuous variable. To study the predictors of physical activity promotion frequency by medical doctors, Multiple Regression Analysis (MRA) was performed, with three blocks of predictors: a first block with sociodemographic variables as independent variables; a second block adding physical activity attitudes, norms and knowledge-related variables; and a third block adding medical doctors’ physical activity levels and sedentary behaviours. Specifically for MRA analysis, nominal and ordinal variables were dichotomized into dummy variables: clinical practice settings [0: hospital healthcare; 1: primary healthcare (continuing care was coded into missing values due to the small number of observations)], medical career position (0: non-specialist; 1: specialist), and medical specialty (0: all other specialties; 1: family medicine). In order to calculate the proportion of explained variance in the dependent variable attributed to each of the considered predictors, part determination coefficients were squared and multiplied by 100. The level of significance was set at *p* ≤ 0.05 for all the analysis, corresponding to a 95% of confidence interval. Previous to the MRA, bivariate analysis (Pearson’s correlation) was also performed between all the variables included in the regression models.

## Results

### Sample description

**Fig. 1:**
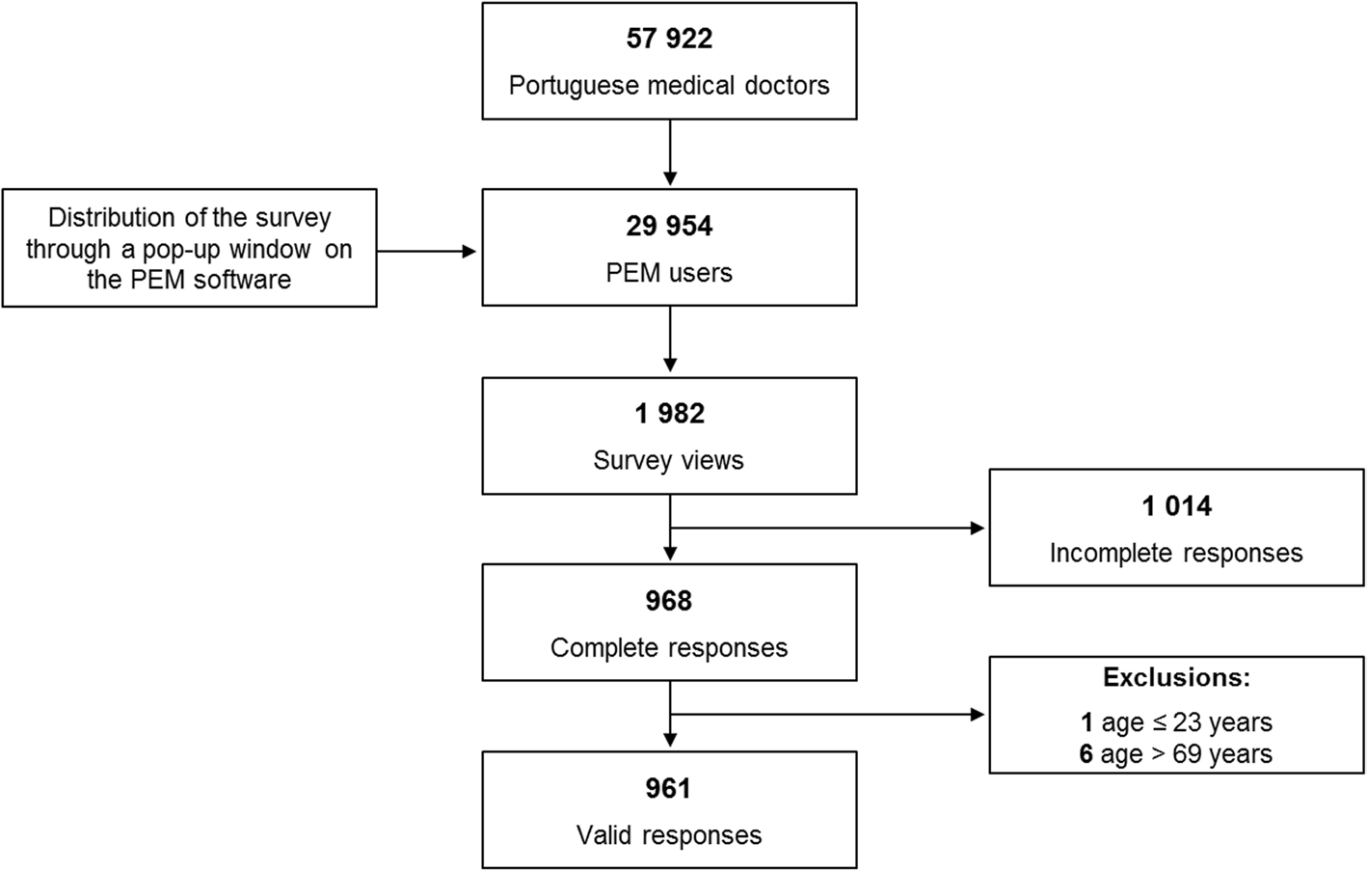
Participants’ flowchart

A total of 1982 medical doctors accessed the survey, which was fully completed by 968 (see Fig. 1 for participants’ flowchart). Seven medical doctors were excluded due to age criterion (one younger than 23 years old; six with more than 69 years). A final sample size of 961 participants was included in the analysis (59% women, mean age 44 ± 13 years), representing 1.7% of overall Portuguese medical doctors and 3.2% of those who have access to the PEM software.

Descriptives for demographic, professional and physical activity-related variables are shown in Table 1. Most participants were specialists (67.2%) and were employed in hospital (53.4%) or primary (45.9%) healthcare settings. The majority (61.8%) attributed a very high level of relevance to physical activity promotion in healthcare and 41.5% stated to have a medium degree of physical activity guidelines knowledge. Although only 35.4% had previous training regarding physical activity promotion, 82.5% had interest in obtain physical activity-related professional training.

**Table 1:** Descriptives for demographic, professional and physical activity-related variables

	***n*** (%)
**Sex**	
Men	398 (41.4)
Women	563 (58.6)
**Age-group**	
23–39 years	471 (49)
40–54 years	205 (21.3)
55–69 years	285 (29.7)
**Medical career position**	
General resident	5 (0.5)
Specialty resident	261 (27.2)
Specialist	646 (67.2)
Non specialist	49 (5.1)
**Clinical practice setting**	
Primary healthcare	441 (45.9)
Hospital healthcare	513 (53.4)
Integrated continuing care	7 (0.7)
**Clinical practice sector**	
Public/National Health Service	691 (71.9)
Private	40 (4.2)
Both	230 (23.9)
**Country’s Health Administrative Region**	
North	300 (31.2)
Centre	181 (18.8)
Lisbon and Tagus Valley	387 (40.3)
Alentejo	46 (4.8)
Algarve	33 (3.4)
Autonomous Region of Azores	1 (0.1)
Autonomous Region of Madeira	13 (1.4)
**Relevance attributed to PA promotion in healthcare**	
Very high	594 (61.8)
High	273 (28.4)
Medium	50 (5.2)
Low	30 (3.1)
Very Low	14 (1.5)
**Perception of request for PA advice from patients**	
Very high	81 (8.4)
High	176 (18.3)
Medium	305 (31.7)
Low	267 (27.8)
Very Low	132 (13.7)
**Perceived knowledge of PA guidelines**	
Very high	61 (6.3)
High	202 (21.0)
Medium	399 (41.5)
Low	224 (23.3)
Very Low	75 (7.8)
**Previous PA training**	
Yes	340 (35.4)
No	621 (64.6)
**Interest in obtaining PA training**	
Yes	793 (82.5)
No	168 (17.5)

*PA* Physical activity

### Physical activity promotion in clinical practice

The frequency of physical activity promotion in clinical practice by sociodemographic and professional groups is shown in Table 2. Most medical doctors (84.6%) reported to promote patients’ physical activity with a medium to a very high frequency (27.7 and 56.9%, respectively), and only 6.6% reported never promoting it. Three sociodemographic characteristics were associated with higher frequencies of physical activity promotion in clinical practice: having a medical specialty (60.8% vs. 51.9% of non-specialists, *p* < 0.05), particularly from family medicine (68.0% vs. 52.1% of medical doctors from other specialties, *p* < 0.001), and working in primary healthcare settings (67.9% vs. 49.3% of medical doctors working in hospital settings, *p* < 0.001).

**Table 2:** Frequency of physical activity promotion in clinical practice by sociodemographic and professional variables

Participants’ characteristics	Frequency of physical activity promotion in clinical practice %				***χ***^**2**^	Cramer’s V	***p*** value
	Never	Very low or low	Medium	High or very high			
**Total**	6.6	7.1	27.7	56.9			
**Sex**							
Men	7.2	8.5	27.2	57.1	2.067	0.047	0.559
Women	6.3	6.3	28.8	58.6			
**Age-group**							
23–39 years	6.0	7.8	30.6	55.6	4.116	0.047	0.661
40–54 years	8.0	7.0	25.0	60.0			
55–69 years	6.8	6.4	26.4	60.4			
**Medical career position**							
Specialist	6.4	6.1	26.6	60.8^a^	7.877	0.091	**0.049**
Non-specialist	7.1	9.4	31.5	51.9^a^			
**Specialty**							
Family medicine	1.4^a^	5.2	25.4	68.0^a^	36.750	0.197	**< 0.001**
Other specialties	9.7^a^	8.4	29.8	52.1^a^			
**Clinical practice setting**							
Primary healthcare	3.4^a^	4.8^a^	23.9^a^	67.9^a^	38.051	0.202	**< 0.001**
Hospital healthcare	9.2^a^	9.4^a^	32.1^a^	49.3^a^			
**Country Region**							
North	7.2	5.8	28.2	58.8	12.418	0.067	0.413
Centre	6.6	7.7	25.4	60.2			
Lisbon and Tagus Valley	5.8	7.3	31.8	55.1			
Alentejo	11.4	11.4	15.9	61.4			
Algarve	9.1	6.1	15.2	69.7			

Differences among Frequency of physical activity promotion categories were tested with Chi-Square tests

^a^|Adjusted standardized residual| > 1.96

When questioned about the main barriers regarding physical activity promotion, lack of perceived interest by patients and lack of time were the two reasons more frequently reported (63.6 and 59.4%, respectively). Lack of technical knowledge and afraid of risks were reported by 36.8 and 10.1% of the participants.

### Predictors of the frequency and content of these clinical practices

Bivariate analysis of the variables included in regression models are presented in Table 3.

**Table 3:** Bivariate correlations between the variables used in the regression models

	1	2	3	4	5	6	7	8	9	10	11	12
**1. PA promotion frequency**	_											
**2. Age**	0.025	_										
**3. Sex**	−0.027	0.102**	_									
**4. Clinical practice setting**	0.193**	−0.006	− 0.061	_								
**5. Medical career position (non-specialists vs. specialists)**	0.072*	0.621**	−0.003	− 0.072*	_							
**6. Specialty (other specialties vs. Family medicine)**	0.190**	−0.071*	− 0.087**	0.790**	− 0.078*	_						
**7. Relevance attributed to PA promotion in healthcare**	0.222**	−0.161**	− 0.076*	0.110**	− 0.123**	0.158**	_					
**8. Patients’ requesting advice on PA**	0.313**	−0.008	−0.054	0.062	0.090**	0.058	0.155**	_				
**9. Perceived knowledge regarding PA guidelines**	−0.072*	−0.085**	0.009	−0.037	−0.070*	− 0.005	0.039	0.006	_			
**10. Previous PA training**	−0.080*	−0.169**	− 0.060	−0.133**	− 0.154**	−0.099**	0.046	0.003	0.349**	_		
**11. Total weekly MET-minutes**	0.118**	0.002	0.072*	−0.076*	−0.031	−0.072*	0.058	0.079*	−0.017	−0.009	_	
**12. Total daily hours of sitting time**	0.054	0.009	−0.017	0.304**	−0.025	0.258**	0.041	−0.005	−0.029	− 0.074*	−0.148**	_

*PA* Physical activity

**p < 0.001; *p < 0.005

Then, three models were tested to analyse predictors of physical activity promotion frequency. Results are presented in Table 4.

**Table 4:** Predictors of physical activity promotion frequency by medical doctors

		Model 1				Model 2				Model 3			
		β (SE)	(95% CI)	***p***	Variance explained (%)	β (SE)	(95% CI)	***p***	Variance explained (%)	β (SE)	(95% CI)	***p***	Variance explained (%)
Constant		3.191 (0.105)	(2.985, 3.397)	**< 0.001**		1.903 (0.205)	(1.500, 2.306)	**< 0.001**		1.804 (0.212)	(1.387, 2.220)	**< 0.001**	
Sociodemogra-phic variables	Age (yrs.)	−0.003 (0.003)	(− 0.008, 0.003)	0.332	0.096	0.000 (0.003)	(−0.006, 0.005)	0.887	0,002	−0.001 (0.003)	(−0.006, 0.004)	0.788	0.006
	Sex	−0.019 (0.058)	(−0.132, 0.094)	0.743	0.011	0.016 (0.054)	(−0.091, 0.122)	0.773	0,007	0.003 (0.054)	(−0.103, 0.109)	0.955	0.000
	Clinical practice setting	0.227 (0.094)	(0.043, 0.411)	**0.015**	0.600	0.185 (0.088)	(0.013, 0.358)	**0.035**	0,395	0.185 (0.088)	(0.011, 0.359)	**0.037**	0.386
	Medical career position (non-specialists vs. specialists)	0.211 (0.078)	(0.059, 0.363)	**0.007**	0.755	0.144 (0.073)	(0.000, 0.288)	**0.050**	0,343	0.159 (0.073)	(0.015, 0.302)	**0.030**	0.415
	Specialty (other specialties vs. Family medicine)	0.173 (0.097)	(−0.017, 0.363)	0.075	0.324	0.124 (0.091)	(−0.054, 0.303)	0.171	0,167	0.128 (0.090)	(−0.049, 0.306)	0.157	0.177
Physical activity norms, attitudes and knowledge-related variables	Relevance attributed to PA promotion in healthcare					0.187 (0.033)	(0.123, 0.251)	**< 0.001**	**2919**	0.181 (0.033)	(0.117, 0.245)	**< 0.001**	**2.708**
	Patients’ requesting advice on PA					0.213 (0.023)	(0.167, 0.259)	**< 0.001**	**7397**	0.206 (0.023)	(0.161, 0.252)	**< 0.001**	**6.907**
	Perceived knowledge regarding PA guidelines					−0.053 (0.028)	(−0.108, 0.002)	0.057	0,325	−0.051 (0.028)	(−0.106, 0.003)	0.065	0.302
	Previous PA training					−0.065 (0.060)	(−0.183, 0.052)	0.275	0,106	−0.061 (0.060)	(−0.178, 0.057)	0.311	0.091
Physical activity levels and sedentary behaviour variables	Total weekly MET-minutes									4.515E-5 (0.000)	(0.000, 0.000)	**0.001**	**1.004**
	Total daily hours of sitting time									0.006 (0.007)	(−0.008, 0.020)	0.385	0.067
F		9.965				21.645				18.948			
*Degrees of freedom*		5				9				11			
R^2^		0.051				0.174				0.184			
R^2^_adjust_		0.046				0.166				0.174			
*p*		**< 0.001**				**< 0.001**				**< 0.001**			

*Abbreviations*: *SE* Standard Error, *CI* Confident Interval, *PA* Physical activity, *MET* Metabolic Equivalents

Model 1, which only included sociodemographic variables (age, sex, clinical practice setting, medical career position, and medical specialty), explained 4.6% (*R*^2^_adj_ = 0.046, *p* < 0.001) of the frequency of physical activity promotion, with two variables associated with a higher frequency of physical activity promotion: working in primary healthcare settings (*p* < 0.05), and having a medical specialty (*p* < 0.05). However, the percentages of explained variance for each of these variables were very low (i.e., less than 1%).

Model 2, where a second block of attitudinal, norms and knowledge-related variables was added (relevance attributed to physical activity promotion in healthcare, patients’ requesting advice on physical activity, perceived knowledge regarding physical activity guidelines, and previous physical activity training), was globally significant, with a higher proportion of the dependent variable explained than the previous one (*R*^2^_adj_ = 0.166, *p* < 0.001). This model included sociodemographic and also physical activity related attitudes, norms and knowledge, and pointed to two extra significant predictors of high frequency of physical activity promotion, in addition to the previously identified ones: attributing a high relevance to the promotion of physical activity in healthcare contexts (*p* < 0.001) and perceiving a high frequency of patients as requesting advice on physical activity (*p* < 0.001).

Model 3, where a third block of medical doctors behaviours was added (total MET-minutes of physical activity per week, and total daily hours of sitting time), was also statistically significant, and the one that better explained the variation of the frequency of physical activity promotion in clinical practice from the three models tested (*p* < 0.001, *R*^2^_adj_ = 0.174). This model included sociodemographic, physical activity-related attitudes, norms and knowledge, physical activity levels and sedentary behaviour. Results highlighted one more significant predictor, besides the previous ones: medical doctors’ total MET-minutes per week of physical activity was associated with a higher frequency of physical activity promotion in clinical practice (*p* = 0.001). Considering all the variables included in model 3, partial proportions of explained variance were more expressive for relevance attributed to physical activity promotion (2.7%) and perception of patients’ requesting advice (6.9%). Medical doctors total MET-minutes per week alone explained 1.0% of the variance of physical activity promotion frequency (Table 4).

### Medical doctors’ physical activity and sedentary behaviours

Table 5 displays the prevalence of physical activity and sitting time by sex, age-group, medical career position, specialty, clinical practice setting, country health region, relevance attributed to physical activity promotion in healthcare, perceived knowledge of physical activity guidelines, and previous physical activity training.

**Table 5:** Medical doctors’ physical activity and sedentary behaviour levels

	Physical activity level						Sitting time					
Participants’ characteristics	Low	Moderate	High	***χ***^***2***^	Cramer’s V	***p value***	< 3 hours/day	> 3 to < 7 hours/day	≥7 hours/day	***χ***^***2***^	***Cramer’s V***	***p value***
**Total**	28.4	45.5	26.1				9.9	25.6	64.5			
**Sex**												
Men	25.9	44.2	29.9	5.481	0.076	0.065	8.8	29.1	62.1	4.794	0.071	0.091
Women	30.2	46.4	23.4				10.7	23.1	66.3			
**Age-group**												
23–39 years	28.0	46.1	25.9	2.502	0.036	0.644	11.0	24.6	64.3	7.667	0.063	0.105
40–54 years	32.2	41.5	26.3				12.7	25.9	61.5			
55–69 years	26.3	47.4	26.3				6.0	27.0	67.0			
**Medical career position**												
Specialist	28.6	45.8	25.5	0.340	0.019	0.844	9.8	27.6	62.7	3.995	0.064	0.136
Non specialist	27.9	44.8	27.3				10.2	21.6	68.3			
**Specialty**												
Family medicine	30.9	46.1	22.9	3.438	0.060	0.179	2.6^a^	13.8^a^	83.^a^	90.788	0.307	**< 0.001**
Other specialties	27.0	45.1	27.9				14.1^a^	32.4^a^	53.6^a^			
**Clinical practice setting**												
Primary healthcare	30.8	44.7	24.5	3.314	0.059	0.191	2.9^a^	13.8^a^	83.2^a^	130.457	0.370	**< 0.001**
Hospital healthcare	25.7	46.6	27.7				15.8^a^	36.1^a^	48.1^a^			
**Country Region**												
North	24.3	47.0	28.7	14.217	0.087	0.076	8.0	28.3	63.7	8.154	0.066	0.419
Centre	31.5	43.1	25.4				7.7	22.7	69.6			
Lisbon and Tagus Valley area	32.0	44.4	23.5				12.1	25.6	62.3			
Alentejo	17.4	45.7	37.0				8.7	19.6	71.7			
Algarve	15.2	60.6	24.2				6.1	27.3	66.7			

*Abbreviations*: *IPAQ* International Physical Activity Questionnaire (short form), *HEPA* Health-Enhancing Physical Activity

Differences among IPAQ categories and sitting time intervals were tested with Chi-Square tests

^a^|Adjusted standardized residual| > 1.96

Regarding physical activity, 26.1% of the sample were classified as “active” (i.e. having a HEPA level), 45.5% were classified as having a “moderate” physical activity level, and 28.4% a “low” physical activity level. No differences were found for sex, age-group, medical career position, medical specialty, clinical practice setting, nor country health region.

The median values obtained for physical activity performed in a usual week, analysed by physical activity level, were as follows: 372.0 (interquartile range [IQR] = 487.5) MET-min.week^− 1^ for “low” level, 1489.5 (IQR = 1024.3) MET-min.week^− 1^ for “moderate” level, and 4320 (IQR = 2205.0) MET-min.week^− 1^ for “active” (HEPA) level.

Regarding sedentary behaviour, 64.5% reported spending, at least, 7 hours per day of sitting time, and only 9.9% reported spending less than 3 hours per day in this behaviour. The median value for total daily hours of sitting time was 8.0 hours per day (IQR = 4.0). Medical doctors from family medicine (83.7% vs. 53.6% of all others *p* < 0.001) and working in primary healthcare settings (83.2% vs. 48.1% working in hospital healthcare settings, *p* < 0.001) spent significantly more time (7 hours per day or above) in sedentary behaviours, both with a strong effect size. Women tend to have higher prevalence than men in the two extreme categories (10.7% vs. 8.8% of men for “< 3 hours/day”; 66.3% vs. 62.1% of men for “≥7 hours/day”), although these differences were not statistically significant (*p* = 0.091). No statistically significant differences for sedentary time intervals were found for age-groups, medical career position, nor country health region.

**Fig. 2:**
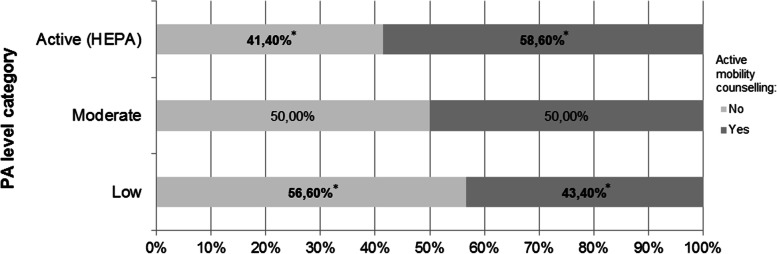
Addressing active mobility in counselling, according to medical doctors’ physical activity levels. Differences between doing or not active mobility counselling were tested with Chi-Square tests. *Statistically significant differences, *p* < 0.05

The content of the counselling provided on physical activity seems to vary according to medical doctors’ own physical activity levels. Addressing the importance of informal physical activity as active mobility, was significantly higher in doctors who had “active” (HEPA) levels of physical activity, when compared with doctors with “low” levels of physical activity [58.6% vs. 43.4%; ***χ***^2^(2, *n* = 898) = 11.396; *p* < 0.005] (Fig. 2).

Having had previous training in physical activity seemed to influence doctors to be more ambitious in their physical activity counselling to patients. Doctors with previous training, when compared with their counterparts with no training, displayed a higher encouragement of their patients to achieve more days of physical activity practice (5 days or more per week) (31.3% vs. 24.3% ***χ***^2^(5, *n* = 898) = 16.257; *p* < 0.05]), and also higher exercise session’ lengths (between 45 and 60 minutes; 22.9% vs. 13.8%; [***χ*** ^2^(5, *n* = 898) = 18.036; *p* < 0.005]).

## Discussion

Concerning the prevalence of physical activity promotion, this study showed that more than half of the sample (56.9%) reported promoting patients’ physical activity with high or very high frequency. A three step model, based on i) sociodemographic variables, ii) attitudes and knowledge-related variables, and iii) physical activity behaviours, was used to explore predictors of promotion frequency. Working in primary healthcare settings, having a medical specialty, attributing a high degree of relevance to patients’ physical activity promotion in healthcare, perceiving a frequent request from patients concerning physical activity counselling, and having higher weekly volume of physical activity were identified as predictors associated with reporting a higher frequency of physical activity promotion counselling practices. Noteworthy, sociodemographic (i.e., clinical practice setting and medical career position) and attitudes-related (i.e., relevance attributed and patients request) variables, identified as predictors in model 1 and 2, remained significant in the subsequent tested model(s), which highlights their independent explanatory power.

Attributing a high degree of relevance to physical activity promotion, and the perception of being frequently requested by patients to counsel on physical activity were the variables showing the highest explanatory power in predicting clinical practices. Additionally, the main barriers were perceived lack of interest by patients, and lack of time. Thus, it seemed that patients’ physical activity counselling occurrence in this sample was more influenced by attitudes (relevance attributed to physical activity counselling) and external factors (i.e., time and patients’ motivation), than for other reasons such as, for example, previous training or lack of technical knowledge.

Although this survey was not developed based on a formal theoretical framework, its outputs are in line with the COM-B model [36]. Briefly, the COM-B model explains behaviour occurrence by identifying three interacting components: 1) individual’s capability (C) to engage in the behaviour; 2) opportunity (O) to prompt the behaviour; and 3) motivation (M) to perform it. These three components need to be in place for a specific behaviour to occur. Applying the four components of the COM-B to our results as a framework for discussion, behaviour occurrence (variable “physical activity promotion frequency by medical doctors”) would be predicted by medical doctors’ capability (or perception of) to perform the behaviour (variable “perceived knowledge regarding physical activity guidelines”), opportunity to implement it (variable “patients’ requesting advice on physical activity”, perceived by the medical doctor as an opportunity to follow with the physical activity promotion), and motivation (variable “relevance attributed to physical activity promotion in healthcare”, as an attitude predisposing medical doctors to promote patients’ physical activity). The results of this study showed that a higher frequency of patients’ physical activity promotion by medical doctors (behaviour occurrence) is predicted, mainly, by a high relevance attributed to patients’ physical activity promotion in healthcare contexts (individual motivational factor) and receiving frequent requesting on physical activity counselling by patients (opportunity). A higher perceived knowledge (perceived capability) was found to be inversely associated with physical activity promotion frequency; however, it was not statistically significant. This may be explained by the fact that more knowledgeable medical doctors’ may not necessarily feel more capable or confident to promote patients’ physical activity. In this regard, it would have been interesting to assess medical doctors’ perceived confidence to promote patients’ physical activity, as a measure of perceived capability, and analyse it as a potential predictor of physical activity promotion frequency. The COM-B model has been identified as a useful model in this area, to put physical activity evidence into practice [37, 38]. It is relevant to note that regression model 3, although statistically significant and the one that better explained the variability of physical activity promotion frequency, still evidenced a low R^2^. Other variables, more related to opportunity, not covered by the questionnaire (the questionnaire only covered medical doctors’ perception of patients motivation/request), as those related to available time and resources, may play an important role in predicting the dependent variable. Specifically, time constraints and perceived lack/availability of opportunities to follow up on advice, local resources to refer patients, and easy-to use and non-time-consuming supporting tools can be of undeniable importance [27]. Other studies analysing the factors associated with physical activity counselling by health professionals also found that opportunities, along with self-efficacy and perceived competence, attitudes and other motivational factors, are unequivocal groups of factors associated with health professionals’ practice [27, 39, 40]. They can act as facilitators or, when absent, as barriers. Identifying and understanding these contextual barriers are fundamental steps when developing a formal physical activity promotion model in healthcare settings. Availability of science-based, user-friendly and non-time consuming tools on physical activity promotion, and training programs for health professionals on how to easily introduce them in their routine care with patients, are a recognized priority in this area [41–43].

Although in this study physical activity knowledge-related variables did not seem to be related to physical activity promotion frequency, they seemed to affect the content of the counselling provided. Participants with previous training on physical activity integrated the prompt for active mobility more frequently in their advice and tended to recommend higher frequencies and durations of physical activity practice to their patients, compared with medical doctors who did not have this background. Previous training supports a more sound, integrated and evidence-based physical activity counselling. In fact, physical activity encompasses not only more planned, formal physical activities – as exercise or sports –, but also a wide range of more informal activities integrated into daily routines [19], predisposing these professionals to rely their counselling on a more inclusive approach of all forms of physical activity, which, perhaps contributes to advice higher week frequencies and lengths of total physical activity.

In alignment with previous literature concerning physical activity promotion in clinical practice [44–46], our study also assessed medical doctors’ own physical activity levels and their influence in these practices. According to 2020 WHO Guidelines on physical activity and sedentary behaviour, adults between 18 and 64 years should do “at least 150–300 minutes of moderate-intensity aerobic physical activity; or at least 75–150 minutes of vigorous-intensity aerobic physical activity; or an equivalent combination of moderate- and vigorous-intensity activity throughout the week” [19]. Results from this survey showed that less than one third of the medical doctors assessed had a level of activity compatible with those guidelines [“active (HEPA)” category]. Indeed, nearly half reported “moderate” and nearly one-third reported “low” physical activity levels. The HEPA levels prevalence found in this sample of medical doctors was very similar to that from a national survey of a representative sample of the Portuguese population (27.1%), also using IPAQ [47]. However, contrary to Portuguese general population data, sex or age-group disparities in physical activity levels were not found in this specific medical doctors sample, nor for other sociodemographic variables. Some previous studies on medical doctors’ physical activity also found a tendency for the absence of differences between men and women or between age groups [48, 49]. This might be explained by the similar lifestyle held by medical doctors and also their socioeconomic status, in a sex and age-independent way.

Regarding sedentary behaviour, the majority (64.5%) reported spending at least 7 hours a day of sitting time. Having this amount of daily sitting time was particularly prevalent in medical doctors from family medicine specialty and/or working in primary healthcare settings. Sitting time behaviour, thus, seems to be more related with the professional context of medical practice than with their physical activity levels, due to the numerous clinical appointments, conferences and administrative work related with patient files [50]. Medical doctors working in primary healthcare, mostly from family medicine specialty, have been pointed by the literature as those who tend to show the worst profile of health behaviours and habits [50, 51], being the second medical specialty that have more recorded extra hours of work in Portugal [52].

Under the tag “Active Doctors, Active Patients”, some scientific evidence has highlighted the positive association between medical doctors’ physical activity level and their clinical practices on physical activity promotion [27, 45, 53], with odds ratio ranging between 1.4 and 5.7, all *p* < 0.05 [53]. The results obtained in our study also showed a positive and statistically significant correlation between physical activity volume and frequency of physical activity promotion. Nevertheless, the regression analysis revealed that doctors physical activity levels only accounted for 1% of the variance on physical activity promotion practices frequency.

This study represents a comprehensive research, given the richness and variability of variables assessed (including physical activity and sedentary behaviour levels) in a large sample of medical doctors working in different settings of a national healthcare system, via a naturalistic survey [i.e. using a national electronic medical software system [54, 55], which contributes to reducing recruitment bias].

Still, it has some limitations that should be mentioned. First, and despite the fact that the survey was disseminated via a national medical software, one can not preclude selection bias, as the ones more predisposed to answer this type of surveys may already have a higher interest on the topic [56]. Selection bias cannot be excluded given that from a total of 29,954 PEM users, only 1982 opened the questionnaire and 968 ultimately submitted the questionnaire fully completed. A low response proportion probably reflects respondents to have a significant different profile (e.g. personal and professional) from non-respondents – for example, more aligned with the topic of physical activity practice or promotion [54]. This may lead to a sample with higher rates of physical activity promotion and physical activity behaviour. Nonetheless, it is important to note, that the present research did not aim to characterize a representative sample of the population of Portuguese doctors. The recruitment method (via medical prescription software) allowed reaching a considerable number of medical doctors in a very time-consuming way, avoiding problems with incorrect/outdated contact information, and limited response duplication, allowing study feasibility. Results found need to be considered keeping in mind the characteristics of the sample recruited. Second, as this is a cross-sectional study, it is not possible to establish direct causal inferences. Results must be carefully interpreted in light of theory-based knowledge and previous research. Third, as this study is based on self-reported measures, participants’ responses may reflect social desirability effects, or interpretation bias to some extent. Nonetheless, concerning physical activity behaviours, IPAQ-SF is a worldwide widely generalized tool used to study physical activity levels of various populations and has demonstrated acceptable validity and reproducibility [57, 58], although without allowing to evaluate medical doctors’ behaviours related to muscle-strengthening activities that are also recommended to fulfil physical activity guidelines. Fourth, data here presented was collected in 2018, not considering possible changes in lifestyles and clinical practices since then. However, it is important to note that one of the main goals of this work was to study the predictors of physical activity promotion frequency, which are likely to remain more stable over time.

### Future directions and practical implications

Future studies in this area can benefit to systematically include the indicators now used, allowing to monitor changes, and to shed light on facilitators and barriers of physical activity promotion practices in healthcare settings, contributing to the development, implementation and testing of interventions in this context. In this regard, 2018 was marked by the launch of the “Global Action Plan for Physical Activity 2018-2030” [5], by the World Health Organization, in which health care systems are highlighted as a crucial setting to promote physical activity, namely through brief counselling implemented by medical doctors and other healthcare professionals. This data was collected a few months before the Global Action Plan launch and, thus, this study may offer an interesting baseline picture for future follow-up studies on the topic of physical activity promotion in the Portuguese health system. Also, informing the development of such studies with theory from the beginning, using theoretical frameworks as the COM-B [36] will strength their scientific outputs and contribute to better explain the processes that determine medical doctors’ clinical practices. Bridging the gap between science and practice is fundamental in public health and to better understand the context in which interventions are planned to be implemented is a key research direction in this area.

In order to implement national policies regarding the establishment of physical activity promotion systems in healthcare settings, the development of formative models that make available appropriate training to healthcare professionals on addressing patients readiness and motivation for physical activity, as well as non-time consuming tools, to support both “easy-to-perform” physical activity assessment and counselling during routine care, must be given attention. The continuous monitoring of these policies will allow for the identification of new implementation needs and adjust further interventions.

## Conclusion

Although less than a third of the medical doctors in this study reported physical activity levels compatible with health benefits (HEPA levels), more than half reported to promote patients’ physical activity with high or very high frequency. Physical activity promotion frequency seems to be higher in primary care settings, in medical doctors having a medical specialty, attributing a higher relevance to physical activity promotion in healthcare, having higher levels of physical activity, and when approached by patients about physical activity.

## Data Availability

The datasets used and/or analyzed during the current study are available from the corresponding author on reasonable request.

## Abbreviations

HEPA: Health-Enhancing Physical Activity
IPAQ: International Physical Activity Questionnaire
MET: Metabolic Equivalents
MRA: Multiple Regression Analysis
NHS: National Health Service
PEM: Electronic Medical Prescription software
SPMS: Shared Services of the Ministry of Health
WHO: World Health Organization
